# Rhythmic auditory cues improve gait asymmetry during unobstructed walking in people with Parkinson’s disease but have no effect on obstacle avoidance - AsymmGait-Parkinson study

**DOI:** 10.3389/fnagi.2025.1455432

**Published:** 2025-02-27

**Authors:** Jônatas Augusto Cursiol, Paulo Cezar Rocha dos Santos, Victor Spiandor Beretta, Diego Orcioli-Silva, Lucas Simieli, Christian Schlenstedt, Daniel Boari Coelho, Fabio Augusto Barbieri

**Affiliations:** ^1^Human Movement Research Laboratory (MOVI-LAB), Department of Physical Education, School of Sciences, São Paulo State University (UNESP), Bauru, Brazil; ^2^Department of Computer Science and Applied Mathematics, Weizmann Institute of Science, Rehovot, Israel; ^3^Center of Advanced Technologies in Rehabilitation, Sheba Medical Center, Ramat Gan, Israel; ^4^Neuroscience and Motor Behavior Laboratory (NEUROCOM-LAB), Department of Physical Education, School of Technology and Sciences, São Paulo State University (UNESP), Presidente Prudente, Brazil; ^5^Posture and Gait Studies Laboratory (LEPLO), Institute of Biosciences, São Paulo State University (UNESP), Rio Claro, Brazil; ^6^Institute of Interdisciplinary Exercise Science and Sports Medicine, Medical School Hamburg, Hamburg, Germany; ^7^Biomedical Engineering, Federal University of ABC, São Bernardo do Campo, Brazil

**Keywords:** walking, rhythmic auditory cueing, rehabilitation, symmetry, neurodegenerative disease, movement disorders

## Abstract

**Introduction:**

This study investigated the influence of rhythmic auditory cues (RAC) on gait asymmetry (GA) during unobstructed and obstacle avoidance walking in people with Parkinson’s disease (PD) and neurologically healthy individuals.

**Methods:**

Thirteen individuals with PD (70.33 ± 6.02 years) and 13 healthy controls (CG) (70.77 ± 7.56 years) participated in this study. They performed a total of five trials during unobstructed walking and 10 trials during obstacle walking under each auditory cue condition (without and with RAC). For obstacle walking, five trials were performed with each limb as leading during obstacle avoidance. First, the volunteers performed the trials without RAC. The trial order, unobstructed or obstacle walking, was randomly defined, and the cues (controlled by a metronome) were personalized according to participants’ cadence. The symmetric index of gait parameters was analyzed using 2 × 2 factorial analysis of variance (group and RAC as factors) separately for each gait type (unobstructed and obstructed walking).

**Results:**

A group-by-auditory cue interaction for step velocity (*p* = 0.027) showed that the PD group exhibited 57.6% reduced asymmetry with RAC during unobstructed walking, with no significant effects observed for the CG. However, RAC had no effect on GA during obstacle avoidance walking in people with PD. Conversely, the CG exhibited 10.5% greater step length asymmetry, 7.1% greater step duration asymmetry, 7.0% greater step velocity asymmetry, and 10.6% greater double support duration asymmetry during obstacle avoidance with RAC (*p* < 0.001).

**Conclusion:**

We conclude that RAC can reduce GA in people with PD during unobstructed walking, but appear to have no effect and negative effects on GA during obstacle walking in people with PD and neurologically healthy individuals, respectively.

## 1 Introduction

Symmetric onset, which refers to the simultaneous or nearly equal appearance of motor symptoms on both sides of the body, is considered a “red flag” for the diagnosis of Parkinson’s disease (PD) (Postuma et al., 2015). Traditionally, motor symptoms on the initially affected side are more severe compared to the other side (Miller-Patterson et al., 2018). This disparity in motor symptom severity contributes to an asymmetric gait (Barbieri et al., 2017). During unobstructed walking, individuals with PD show increased stride time, swing time (Patoz et al., 2023) and step length asymmetry (Seuthe et al., 2024) compared to neurologically healthy controls. Asymmetry also manifests when crossing an obstacle with asymmetry patterns in the leading and trailing toe clearance (Orcioli-Silva et al., 2020). In fact, obstacle avoidance requires heightened sensorimotor integration, leading to reduced automatic control due to the absence of cognitive compensatory options (Vitório et al., 2023). This increases gait asymmetry compared to unobstructed walking in people with PD (Barbieri et al., 2018a). Although not entirely understood, the asymmetric neural dysfunction in the basal ganglia (Riederer and Sian-Hülsmann, 2012; Van der Hoorn et al., 2011) and the asymmetrical onset of motor symptoms, such as bradykinesia, rigidity and postural control (Peterson and Horak, 2016), may contribute to gait asymmetry in people with PD.

Pharmacological treatment is widely implemented to decrease PD signs and symptoms and also seems to be effective in reducing gait asymmetry (Barbieri et al., 2018a; Warmerdam et al., 2021; Wilson et al., 2020). Barbieri et al. (2018a) showed that antiparkinsonian medication reduced both stride length asymmetry and stride velocity asymmetry during obstacle circumvention in people with PD. However, prolonged use of dopaminergic medication may cause dependency, side effects (e.g., dyskinesia), and reduce the medication’s effectiveness (Olanow and Stocchi, 2018). The effect of medication tends to decrease throughout the years with the progression of the disease, leading to gait impairments such as reduced step length, decreased walking speed, and increased variability in stride time (Wilson et al., 2020). Additionally, Warmerdam et al. (2021) indicated that walking at higher speeds or avoiding obstacles may reduce the effectiveness of dopaminergic medication in people with PD. These conditions increase the complexity of motor control demands, potentially surpassing the compensatory effects of the medication. Hence, complementary interventions have been explored to decrease gait asymmetry, such as the use of additional externally driven stimuli.

Previous studies indicated that rhythmic auditory cues (RAC), defined as external rhythmic stimuli, such as metronome beats or music with a consistent tempo, are a complementary and effective intervention for counteracting PD-related gait impairments (Forte et al., 2021; Ghai et al., 2018; Rinaldi et al., 2019). RAC complement drug treatment for PD by using the constant beat of a metronome or music to improve balance and gait problems (Ginis et al., 2018; Pando-Naude et al., 2024), resulting in improvements in walking velocity and stride length (Belluscio et al., 2021; Minino et al., 2021). Additionally, Rinaldi et al. (2019) observed that RAC could increase muscle activation, potentially improving stability and aiding individuals with PD to safely cross obstacles. Particularly relevant, the use of RAC may minimize PD-related impairments in gait control, reflecting improved obstructed walking performance through greater propulsive braking impulses and shorter time to process obstacle characteristics (Rinaldi et al., 2019), thereby reducing the risk of falls.

From a neurological perspective, RAC’s effects on regulating gait are likely related to improvements in brainstem and cerebellum activations (Devlin et al., 2019), reducing deficits involved in automatic movement in the basal ganglia (Koshimori and Thaut, 2018), and providing phasic cues to the supplementary motor area (Woerd et al., 2017). Prior studies have employed diverse methodologies to apply RAC, such as metronome beats at individualized cadences (Devlin et al., 2019) or music with embedded rhythmic patterns (Koshimori and Thaut, 2018). These stimuli were typically synchronized with participants’ steps to enhance temporal gait parameters, with variations in time, to examine effects during both steady-state and obstacle-crossing scenarios. Our experimental design builds on these findings by specific justification, e.g., testing novel rhythmic patterns, focusing on obstacle negotiation, or exploring underrepresented gait metrics, allowing for a comprehensive evaluation of RAC’s effects on both obstructed and unobstructed walking. Brodie et al. (2015) found that symmetry-matched RAC compensated for unsteady gait in most people with PD without exacerbating gait asymmetry. However, the authors suggested that RAC may be particularly beneficial in cognitively demanding walking tasks, such as obstacle avoidance, which require precise motor coordination and significant attentional resources. Based on this evidence, we hypothesize that RAC will reduce gait asymmetry (e.g., decrease step length and velocity asymmetry) during obstacle avoidance walking, where its effects are more likely to manifest, but not during unobstructed walking, where motor demands are comparatively lower.

The purpose of this study is to investigate whether auditory cueing can reduce gait asymmetry during unobstructed walking and obstacle avoidance in people with PD. The novelty of this study lies in testing the effects of RAC on gait asymmetry during obstacle avoidance in people with PD and matched-control individuals. Understanding how people with PD interact with sensory information during walking is crucial in order to make clinical decision-making for personalized care and design assistive devices.

## 2 Materials and methods

### 2.1 Participants

The analysis with G*Power software (version 3.1; University of Düsseldorf, Dusseldorf, Germany) showed that a sample size of at least 26 individuals (13 in each group) would be necessary to obtain a power of 80% probability to detect a difference of 20% between the two groups for the primary outcome with a type I error of 0.05, based on previously published data (Rinaldi et al., 2019). Hence, a total of 13 individuals with PD (PD group - seven females) and 13 neurologically healthy participants (CG) (eight females) matched by age, height, and body mass participated in this study. Participants with PD and neurologically healthy control participants were recruited from the local community and rehabilitation programs. All participants underwent an interview to collect demographic, sociocultural, and overall health information. An experienced neurologist evaluated and confirmed the diagnosis of PD specifically for this study using the London Brain Bank criteria (Hughes et al., 1992). This evaluation included a detailed clinical assessment and a comprehensive review of medical history to ensure diagnostic accuracy and participant eligibility. Neurologically healthy individuals were screened to ensure the absence of any neurological or motor impairments.

The inclusion criteria were: independent living in the community; ability to walk without the use of any aids; absence of uncontrolled diabetes, hypertension, cardio-respiratory diseases, balance, and vision disorders that may impair locomotion. In addition, for the PD group, the individuals were included in the study if they had a diagnosis of idiopathic PD, were taking their antiparkinsonian medication regularly and with a Hoehn & Yahr stage (H&Y) (Hoehn and Yahr, 1967) 3 or below. Participants with PD were evaluated in the ON-state of medication (1 h after taking their dopaminergic medication).

### 2.2 Clinical evaluations and determination of footedness of the control group and most affected limb for individuals with PD

Initially, an experienced evaluator conducted an anamnesis to characterize both the PD and control groups, focusing particularly on the side where PD symptoms were present. After that, a clinical assessment using the motor section of the Unified Parkinson’s Disease Rating Scale (UPDRS) (Fahn and Elton, 1987; Goetz et al., 2003) and the H&Y scale was applied to determine the motor disease severity and the stage of disease of PD participants, respectively. In addition, cognitive condition screening in both groups was analyzed using the Mini-Mental State Examination (MMSE) (Brucki et al., 2003).

Footedness was assessed in the CG by asking all participants to kick a ball to hit a target. The limb that each individual preferred to kick the ball was considered as the preferred limb. For PD participants, motor UPDRS items 20–23 and 25–26 were used to assess appendicular asymmetry (Araújo-Silva et al., 2022). The most affected limb was determined by calculating the difference between the scores of the right and left limbs in the UPDRS items mentioned above. A positive value indicated the right limb as the most critically affected, whereas negative values indicated the left limb.

### 2.3 Gait asymmetry evaluation with and without rhythmic auditory cues

Participants performed four walking conditions: unobstructed gait without and with RAC, and obstacle avoidance without and with RAC. A total of five trials were performed in each auditory cue condition for unobstructed walking, and 10 trials were performed in each auditory cue condition for obstacle avoidance - five trials for each limb crossing the obstacle. First, participants performed a trial without RAC. The order of trial conditions was randomly assigned, comprising unobstructed trials, obstacle avoidance trials with the most affected/non-preferred limb, and obstacle avoidance trials with the least affected/preferred limb. The same sequence was maintained for the trials conducted with and without RAC.

**FIGURE 1:**
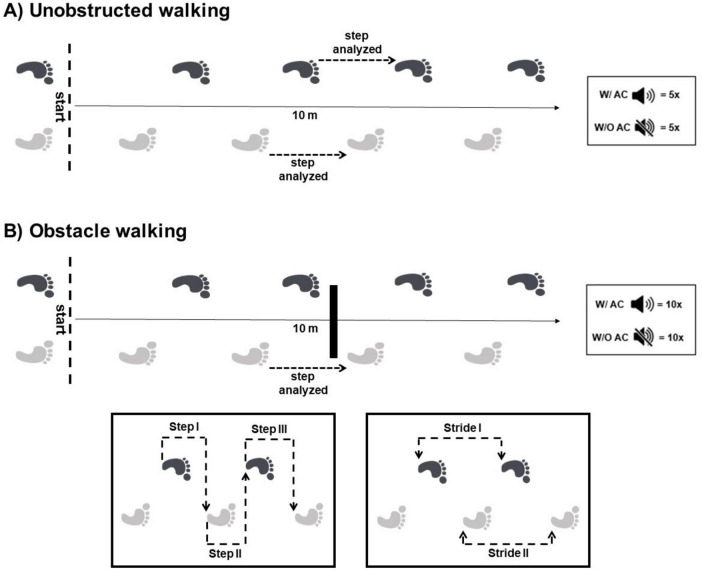
Representative illustration of the steps analyzed in unobstructed walking **(A)** and obstacle avoidance **(B)**. Obstacle features: 15 cm height × 80 cm width × 2 cm thick. W/ AC, with auditory cue; W/O AC, without auditory cue. A total of five trials in each cue condition were performed in unobstructed walking and 10 trials, five for each limb crossing the obstacle in obstacle walking. The crossing step was analyzed when the leading limb crossed the obstacle.

Participants were instructed to walk at their self-selected pace with the instruction, “Please walk at your preferred speed, just like you would on the street,” to maintain uniformity across trials. Walking took place on a 10-m wooden pathway covered with a 3 mm thick black rubber carpet. During the obstacle avoidance trials, participants were instructed to avoid contact with a single obstacle (15 cm high, 80 cm wide, and 2 cm thick) placed midway at the 5-m mark. The starting point was adjusted to ensure participants completed at least two strides before encountering the obstacle, ensuring consistency in task complexity and conditions for all participants. The experimental design is demonstrated in Figure 1.

For auditory cue trials, the cues were personalized for each individual according to the cadence determined by three unobstructed trials performed prior to the start of the experiment. During auditory cue trials, participants were instructed to walk normally, while stepping in time with the metronome beats, trying to synchronize each heel contact with the beat. Participants performed two trials to practice before starting. The trials were stopped and repeated if participants ceased to follow the cues during the experiment.

RAC were delivered using a metronome application on a smartphone, played through a speaker placed at a comfortable distance from the participants. The participants listened to the cues during the walking trials through the speaker, and the volume was adjusted individually to ensure that the cues were clearly audible but not overwhelming. The auditory cues were provided continuously throughout the walking task, starting at the beginning of the trial and stopping once the participant had completed the walking task. The tone of the metronome was consistent throughout, and the frequency was maintained at the specified beats per minute for each group.

### 2.4 Data acquisition

The acquisition of kinematic gait parameters was accomplished using a GAITRite^®^ system (CIR System, Clifton, NJ, USA) with a sample rate of 200 Hz. For unobstructed gait, one step from each side of the middle of the pathway was analyzed per trial. Steps were taken from the middle of the 10-m pathway, defined as the section between 2.5 and 7.5 m. This segment was selected to capture steady-state walking, minimizing the influence of acceleration and deceleration phases. The same length was used for all participants to ensure uniformity in the data collection process.

For obstacle avoidance, the crossing step taken by the leading limb during the avoidance maneuver was analyzed in each trial. Length, duration, width, velocity, limb swing duration (as a percentage of step duration), and double support duration (as a percentage of step duration) were calculated for each step.

To investigate the gait asymmetry, we considered the steps with the most and least affected limb (right or left according to UPDRS items) for PD participants as well as preferred and non-preferred limb (right or left according to footedness test) for the control group in each gait type. Although a recent study by Seuthe et al. (2024) suggests a potential mismatch between step length dominance and UPDRS-based dominance, our classification was solely used to ensure consistent averaging for the same leg across multiple trials.

Initially, the average of each gait parameter was calculated for the most and least affected limb in PD participants, and for the preferred and non-preferred limb in the control group. Next, the Symmetry Index (SI) (Herzog et al., 1989) was calculated for each participant based on gait conditions, where MA represents the most affected limb, P is the preferred limb, LA is the least affected limb, and NP is the non-preferred limb. A value of zero for an index indicates no difference between the sides. Since we were not interested in the side effect of asymmetry but in the effect of RAC, we adopted the absolute value of the SI for the statistical analysis. For obstacle avoidance, the average of each gait parameter of the crossing step with the most and least affected limb, or preferred and non-preferred limb, was used to calculate the SI.


S⁢I=

$$\left[\frac{\left(v\,a\,l\,u\,e\,o\,f\,M\,A\,o\,r\,P\,l\,i\,m\,b-v\,a\,l\,u\,e\,o\,f\,L\,A\,o\,r\,N\,P\,l\,i\,m\,b\right)}{\left(v\,a\,l\,u\,e\,o\,f\,M\,A\,o\,r\,P\,l\,i\,m\,b+v\,a\,l\,u\,e\,o\,f\,L\,A\,o\,r\,N\,P\,l\,i\,m\,b\right)}\right]\times 100\%$$

### 2.5 Statistical analysis

Statistical analyses were performed using the JASP (version 0.18.3) for Windows. Normality and homogeneity were checked through the Shapiro-Wilk and Levene’s tests, respectively. To analyze cognitive status, a Student’s *t*-test for independent samples was employed to compare the PD group with the control group. Additionally, this test was conducted to evaluate potential differences in cadence between the groups under the RAC condition. To analyze the symmetric index of gait parameters, a 2 × 2 factorial analysis of variance (ANOVA) with independent measures was performed, considering the factors “Group” (PD group vs. CG) and “RAC” (with vs. without auditory stimulus). These analyses were conducted separately for unobstructed walking and obstacle-avoidance walking conditions. Post hoc analyses using Tukey’s tests were conducted to explore significant effects. Cohen’s d was used to report the effect size for the Student’s *t*-test comparing cognitive status between the PD group and the control group, while Eta squared (η^2^) was used to report effect sizes for the ANOVA results. Statistical significance for all analyses was set at *p* < 0.05, with effect sizes interpreted as Cohen’s d (>0.2 small effect; >0.5 medium effect; >0.8 large effect) or η^2^ as small (effect size > 0.01), moderate (effect size > 0.06), or large (effect size > 0.14) (Cohen, 1988).

## 3 Results

The group characteristics and clinical variables are detailed in Table 1. Cognitive levels, measured by the Mini Mental State Examination, were comparable between the PD group and CG (t_24_ = 1.95; *p* = 0.059, *d* = 0.783). The average frequencies were 106.92 beats per minute for the PD group and 109.07 beats per minute for the CG.

**TABLE 1:** Means and standard deviations of characteristics and clinical variables for individuals with PD and the control group.

Variable	PD group	CG	Min-max (PD)	Min-max (CG)	*p*-value	Cohen’s *d*
Age (years)	70.33 ± 6.02	70.77 ± 7.56	61–80	59–81	0.869	0.065
Height (m)	1.62 ± 0.08	1.60 ± 0.06	1.50–1.70	1.50–170	0.784	−0.109
Body mass (kg)	68.20 ± 12.48	70.15 ± 10.32	42.00–82.80	56.40–83.50	0.667	0.171
MMSE (pts)	26.85 ± 1.82	28.38 ± 2.10	23–30	24–30	0.058	0.783
UPDRS III ON (score)	24.08 ± 6.18	–	14–35	–	–	–
H&Y (score)	2.08 ± 0.34	–	1.5–2.5	–	–	–
CUW-RAC (steps/min)	107.37 ± 12.75	113.01 ± 12.73	83.14–132.18	90.88–136.18	0.270	0.443
COW-RAC (steps/min)	80.93 ± 10.75	89.21 ± 10.57	65.80–106.08	68.95–104.38	0.059	0.777

MMSE, Mini Mental State Examination; UPDRS III ON, motor section of the Unified Parkinson’s disease rating scale conducted when the participant was in the “ON” state of medication; H&Y, Hoehn & Yahr; CUW-RAC, Cadence during unobstructed walking with RAC; COW-RAC, Cadence during obstacle-avoidance walking with RAC; *p*-values marked in bold represent statistically significant results.

Means and standard deviations of kinematic gait parameters under each condition (unobstructed vs. obstacle walking), by group (PD vs. CG), side (preferred vs. non-preferred), and cue presence (with vs. without RAC), are presented in Supplementary Tables 1–6. The ANOVAs revealed statistically significant differences in step length, duration, width, velocity, limb swing duration, and double support duration between PD and control groups across walking conditions and limb preferences.

### 3.1 Unobstructed walking

**FIGURE 2:**
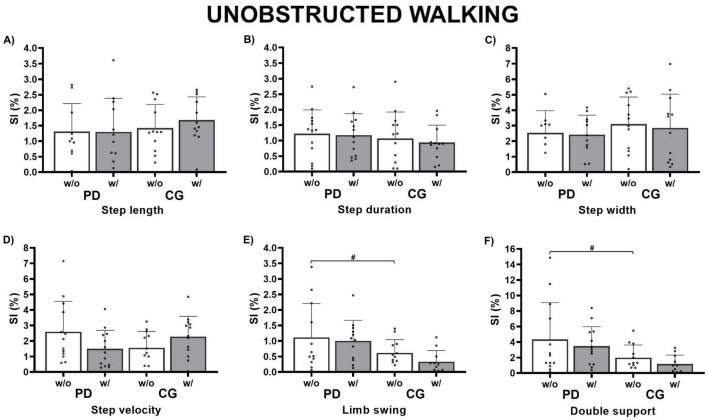
Bar graphs showing the results of the Group × RAC interaction for the unobstructed walking condition. Circles represent individual values. #Indicates significant differences identified in post-hoc comparisons between the groups (PD vs. CG) without auditory cues. SI (%), symmetry index; w/o, without auditory cue (white); w/, with auditory cue (gray); PD, Parkinson’s disease group; CG, control group. Panel **(A)** Step length; **(B)** Step duration; **(C)** Step Width; **(D)** Step velocity; **(E)** Limb swing; **(F)** Double support.

Table 2 presents the symmetry index of the spatial-temporal parameters. The ANOVAs revealed a main effect of group for limb swing [F(1, 26) = 4.013; *p* = 0.007, η^2^ = 0.16] and double support duration [F(1, 26) = 7.728; *p* = 0.008, η^2^ = 0.14], demonstrating greater asymmetry (44.8% and 40.4% higher, respectively) in the PD group compared to the CG (Figure 2). Furthermore, a group-by-auditory cue interaction for step velocity [F(1, 26) = 5.193; *p* = 0.027, η^2^ = 0.10] showed that the PD group exhibited 57.6% reduced asymmetry with RAC, with no significant effects observed for the CG.

**TABLE 2:** The means and standard deviations of the symmetric index (%) of walking parameters in the PD group (PD group) and control group (CG) are presented for unobstructed and obstacle avoidance walking, both without and with auditory cues.

	PD group		CG		Effects of group	Effects of auditory cue	Effects of interaction
**Symmetric index (%)**	**Without cues**	**With cues**	**Without cues**	**With cues**			
**UNOBSTRUCTED WALKING**							
Step length	1.31 ± 0.91	1.30 ± 1.09	1.42 ± 0.77	1.68 ± 0.75	0.350 (0.02)	0.643 (0.01)	0.616 (0.01)
Step duration	1.23 ± 0.77	1.17 ± 0.70	1.07 ± 0.86	0.94 ± 0.56	0.345 (0.02)	0.661 (0.00)	0.849 (0.00)
Step width	2.54 ± 1.43	2.42 ± 1.26	3.10 ± 1.76	2.85 ± 2.19	0.350 (0.02)	0.734 (0.00)	0.901 (0.00)
Step velocity	2.60 ± 1.96	1.50 ± 1.19	1.56 ± 1.05	2.29 ± 1.30	0.760 (0.00)	0.639 (0.00)	**0.027** (0.10)
Limb swing duration	1.12 ± 1.09	1.00 ± 0.66	0.62 ± 0.43	0.33 ± 0.36	**0.007** (0.16)	0.336 (0.02)	0.672 (0.00)
Double support duration	4.33 ± 4.74	3.49 ± 2.48	1.99 ± 1.63	1.17 ± 2.48	**0.008** (0.14)	0.328 (0.02)	0.991 (0.00)
**OBSTACLE WALKING**							
Step length	1.59 ± 1.04	2.34 ± 1.36	2.28 ± 1.69	5.74 ± 3.92	**0.003** (0.14)	**0.002** (0.15)	**0.043** (0.06)
Step duration	2.30 ± 1.67	1.42 ± 1.37	1.96 ± 0.68	6.29 ± 4.13	**0.001** (0.15)	**0.012** (0.09)	**<0.001** (0.21)
Step width	3.80 ± 2.76	7.60 ± 5.24	7.57 ± 5.85	9.77 ± 9.31	0.104 (0.06)	0.102 (0.06)	0.657 (0.00)
Step velocity	2.20 ± 1.55	3.18 ± 2.50	2.48 ± 1.53	8.13 ± 3.93	**<0.001** (0.15)	**<0.001** (0.25)	**0.002** (0.12)
Limb swing duration	0.36 ± 0.26	0.32 ± 0.23	0.82 ± 0.53	1.22 ± 0.76	**<0.001** (0.31)	0.227 (0.02)	0.148 (0.03)
Double support duration	2.04 ± 1.40	1.98 ± 1.89	3.43 ± 1.85	7.62 ± 3.85	**<0.001** (0.27)	**0.007** (0.09)	**0.006** (0.10)

Effect size - η^2^ (eta squared); *p*-values marked in bold represent statistically significant results. The final three columns display the significance values and effect sizes (in parentheses) for the main effects of group and auditory cue, as well as the group × auditory cue interaction.

### 3.2 Obstacle avoidance walking

**FIGURE 3:**
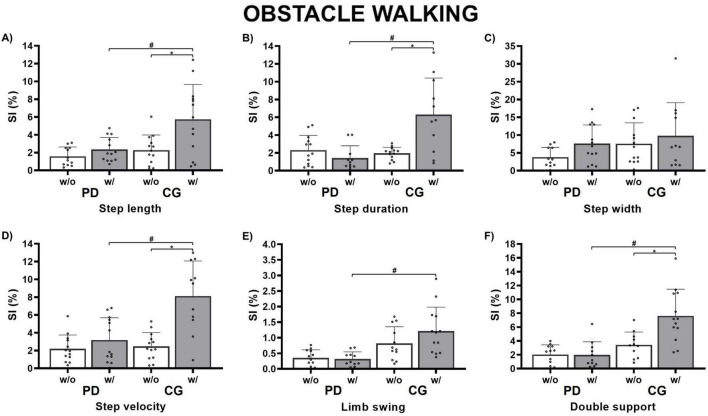
Bar graphs showing the results of the Group × RAC interaction for the obstacle avoidance walking condition. Circles represent individual values. #Indicates significant differences identified in post-hoc comparisons between the groups (PD vs. CG) with auditory cues. *Denotes the main effect of the auditory cue within the group. SI (%), symmetry index; w/o, without auditory cue (white); w/, with auditory cue (gray); PD, Parkinson’s disease group; CG, control group. Panel **(A)** Step length; **(B)** Step duration; **(C)** Step Width; **(D)** Step velocity; **(E)** Limb swing; **(F)** Double support.

The presence of obstacles significantly increased the asymmetry scores for both PD and control groups (Figure 3). A main effect of group was found for step length [F(1, 26) = 9.93; *p* = 0.003, η^2^ = 0.14], step duration [F(1, 26) = 11.75; *p* = 0.001, η^2^ = 0.15], step velocity [F(1, 26) = 13.68; *p* < 0.001, η^2^ = 0.15], limb swing duration [F(1, 26) = 5.575; *p* < 0.001, η^2^ = 0.31], and double support duration [F(1, 26) = 23.06; *p* < 0.001, η^2^ = 0.27], indicating that the CG exhibited significantly greater asymmetry compared to the PD group (step length: 59%, step duration: 47%, step velocity: 56%, limb swing duration: 12%, and double support duration: 23% higher, respectively).

A main effect of auditory cue was found for step length [F(1, 26) = 10.54; *p* = 0.002, η^2^ = 0.15], step duration [F(1, 26) = 6.852; *p* = 0.012, η^2^ = 0.09], step velocity [F(1, 26) = 21.94; *p* < 0.001, η^2^ = 0.25], and double support duration [F(1, 26) = 8.004; *p* = 0.007, η^2^ = 0.09, moderate], indicating that the CG showed more asymmetry with RAC compared to without RAC (step length: 10.5%, step duration: 6.9%, step velocity: 21.9%, and double support duration: 8.0% higher, respectively).

ANOVAs revealed a group-by-auditory cue interaction for step length [F(1, 26) = 4.325; *p* = 0.043, η^2^ = 0.06], step duration [F(1, 26) = 15.58; *p* < 0.001, η^2^ = 0.21], step velocity [F(1, 26) = 10.93; *p* = 0.002, η^2^ = 0.12], and double support duration [F(1, 26) = 8.468; *p* = 0.006, η^2^ = 0.10]. During the condition with RAC, the CG showed 10.9% greater step length asymmetry, 6.9% greater step duration asymmetry, 21.9% greater step velocity asymmetry, and 8.5% greater double support duration asymmetry than the PD group (*p* < 0.001). Furthermore, the CG exhibited 10.5% greater step length asymmetry, 7.1% greater step duration asymmetry, 7.0% greater step velocity asymmetry, and 10.6% greater double support duration asymmetry during obstacle avoidance with RAC compared to without RAC (*p* < 0.001), with no significant effects observed for the PD group.

## 4 Discussion

We investigated whether rhythmic auditory cues reduce gait asymmetry during unobstructed and obstacle avoidance walking in people with PD and neurologically healthy individuals. Interestingly, our hypothesis was only partially supported. Although RAC decreased step velocity asymmetry during unobstructed walking in people with PD, they had no effect on gait asymmetry during obstacle avoidance walking. Additionally, one unexpected finding was that RAC during obstacle avoidance increased asymmetry in step length, duration, velocity, and double support duration in neurologically healthy individuals. Our results extend the current literature, suggesting that RAC represent an effective strategy to reduce gait asymmetry in people with PD during unobstructed walking, without affecting gait asymmetry during obstacle avoidance. These findings may have significant implications for planning gait interventions in people with PD.

### 4.1 Rhythmic auditory cues reduce step velocity asymmetry during unobstructed walking in people with PD

RAC significantly improved gait asymmetry in people with PD during unobstructed walking, notably reducing step velocity asymmetry (η^2^ = 0.10, moderate). Our findings are in line with previous research and highlight the effectiveness of RAC in improving gait in people with PD. RAC, utilized through a metronome or via music, have been recommended for reducing gait impairments in people with PD (Forte et al., 2021; Ghai et al., 2018; Rinaldi et al., 2019), potentially enhancing balance (Pando-Naude et al., 2024), walking speed, and stride length (Belluscio et al., 2021; Minino et al., 2021) by increasing muscle activation (Rinaldi et al., 2019) during unobstructed walking. The key neurophysiological mechanism behind this effectiveness is that external cues can redirect neural circuits from those that are more affected to those that are not, leading to a shift from habitual to goal-directed (Redgrave et al., 2010). RAC, in particular, perform an executive role in facilitating attention focus on gait and a stabilizing role in reducing variability while improving spatiotemporal gait parameters (Ginis et al., 2018). Moreover, RAC can also reduce gait asymmetry in individuals with PD by enhancing external timing, motor planning, and sensory integration (Ghai et al., 2018). Previous theories suggest that RAC may act as an external generator, optimizing motor execution by recalibrating predictions based on sensory consequences (Harrison and Earhart, 2023; Izawa et al., 2012).

Using RAC can be a beneficial strategy for dealing with the asymmetric neural neurodegeneration seen in PD. It induces functional asymmetry between hemispheres, with a relative reduction of neural excitability in the most affected hemisphere and an apparent increase in the least affected one (Claassen et al., 2016; Riederer and Sian-Hülsmann, 2012), as well as reduced transcallosal sensorimotor structural connectivity (Fling et al., 2018). In a previous study of our group, we showed that people with PD presented asymmetric cortical activity behavior during gait initiation, with no asymmetry in anticipatory postural adjustments (Faria et al., 2023). We interpreted that higher asymmetry in cortical activity was an adaptive mechanism to improve motor behavior (specifically, gait initiation), as damaged areas of the most affected hemisphere are substituted by residual networks within both hemispheres (Berenguer-Rocha et al., 2020; Brain et al., 2020; Knights et al., 2021; Peterson and Fling, 2018). Therefore, in the current study, we suggest that RAC can also have a positive impact on brain activity, functionally remapping from degenerated areas onto homologous areas within the hemisphere experiencing less degeneration (Berenguer-Rocha et al., 2020). Additionally, RAC are likely to enhance the activity of the brainstem and cerebellum (Devlin et al., 2019), mitigating deficits related to automatic movement control in the basal ganglia (Koshimori and Thaut, 2018), providing temporal cues to the supplementary motor area (Woerd et al., 2017), and consequently reducing gait asymmetry.

The effect on step velocity is intriguing as it encompasses both the spatial and temporal aspects of gait. Gait velocity is determined by the ratio of step length to step duration. Thus, one may argue that a reduction in step velocity asymmetry can indicate a decrease in either step length asymmetry, step duration asymmetry, or both. While step velocity asymmetry tends to worsen with age to compensate for the reduction in step length (Forte et al., 2021), our study demonstrates that RAC can be effective in promoting gait symmetry in people with PD during unobstructed walking. These findings offer novel insights for gait interventions in people with PD.

### 4.2 Rhythmic auditory cues do not change gait asymmetry in PD, but increase asymmetry in healthy individuals during obstacle avoidance

RAC did not affect obstacle avoidance during walking in people with PD. Therefore, it seems that RAC do not interfere with gait asymmetry during obstacle avoidance in people with PD. Obstacle avoidance represents a challenging task that is likely to increase gait asymmetry in both people with PD and neurologically healthy individuals (Barbieri et al., 2018b). Although previous studies have shown that RAC during obstacle avoidance walking benefit both people with PD and neurologically healthy individuals to safely overcome obstacles due to better positioning of the feet on the ground, stability and ability to maintain synchronization with RAC (Rinaldi et al., 2019), our findings indicated that they do not appear to have an effect on gait asymmetry.

One possible explanation is that due to the increase in difficulty, complexity, motor cortex activity and cognitive resources required during obstacle avoidance (Ambike et al., 2021), PD participants probably prioritized the motor task and ignored the external cues, increasing the attention control to perform obstacle avoidance. A possible additional explanation may be related to the weight of sensory information attributed to vision. According to Faria et al. (2023), obstacles up to 15 cm high, such as the one used in our study, act as a visual cue during the gait of people with PD. In this case, visual information is utilized to track the characteristics of the obstacle in order to make adjustments to safely prevent it (Santos et al., 2010). Furthermore, Föcker et al. (2022) elucidated that the visual signal can make it difficult to integrate an auditory cue. Therefore, due to the risk and importance of spatial variables when negotiating with an obstacle to avoid tripping, visual information may have had more relevance/weight compared to auditory information. However, our findings should not be interpreted negatively. On the contrary, these results may serve as helpful indicators for implementing the RAC approach in gait intervention, considering that RAC do not interfere with gait asymmetry and promote other benefits in gait during obstacle avoidance in people with PD.

An intriguing finding of this study is that, for certain gait parameters such as step velocity and step length the PD group exhibited lower asymmetry values than the control group during obstacle avoidance walking, particularly in the absence of RAC. One possible explanation for this observation is that individuals with PD are more accustomed to managing motor deficits and may inherently adopt compensatory mechanisms to maintain balance and motor control, even without external cues. One important aspect here is that our sample is composed of adults over 65 years old, which may be minimally affected by the aging process in motor and non-motor variables. It may suggest that the gait pattern is disrupted during obstacle avoidance in older adults, likely increasing gait variability (Baker et al., 2008; Harrison and Earhart, 2023). Harrison and Earhart (2023), in a recent systematic review, found similar findings for gait variability during rhythmic RAC: from 11 papers with neurologically healthy older adults, 10 papers found no or negative (higher) effects on gait variability. The authors suggest that for healthy older adults with minimal gait impairment, RAC may disrupt the normal gait pattern, potentially relating to increased attentional or cognitive demands in order to synchronize gait to an outside source.

The increase in gait asymmetry in the CG can be explained by the fact that it is more challenging for older adults to synchronize their gait with a rhythm as it is an action that can lead to dual-task interference that slows gait and shortens steps (Roberts et al., 2021). Very likely, RAC during obstacle avoidance increase the robustness of the task, which may interfere with well-learned movement patterns in people without basal ganglia deficits and perturb the functioning of internal mechanisms (Brodie et al., 2015). Nevertheless, the study’s major limitation was the lack of specificity in determining the metronome pace for obstacle walking, potentially causing discrepancies between unobstructed and obstacle situations. This may have interfered with the obstacle walking results. Future studies should confirm auditory cue frequency separately for each condition. It is possible that the CG walked faster during unobstructed walking, which may have been too fast to maintain during obstacle avoidance, leading to increased asymmetry. The CG might have adjusted their gait rhythm during obstacle avoidance, possibly changing the rhythm only on the non-preferred side, contributing to gait asymmetry.

Other limitations of our study should be mentioned. First, participants were restricted to those with mild to moderate PD. As demonstrated by Lirani-Silva et al. (2019), the benefits of RAC increase as diseases progress. Future research could include people with more severe PD (H&Y ≥ 3), who experience freezing of gait with and without medication, and those with greater gait asymmetry. Furthermore, gait parameters did not demonstrate any significant changes in the auditory cue condition. Although we observed significant changes in certain gait parameters, such as step velocity asymmetry during unobstructed walking in the PD group, other parameters like step length and step duration did not demonstrate significant changes in response to RAC. This suggests that RAC may not uniformly affect all gait parameters and highlights the complexity of gait modulation in different walking conditions. Even though a single session of auditory cue administration may improve gait asymmetry in people with PD (Belluscio et al., 2021), familiarization and longer administration of auditory cue training could be necessary to observe significant improvements in spatiotemporal gait parameters in PD.

## 5 Conclusion

We conclude that even though RAC demonstrate a beneficial effect on gait asymmetry during unobstructed walking in people with PD, their impact on gait asymmetry during obstacle avoidance is negligible in this population. Interestingly, in neurologically healthy older adults, RAC during obstacle avoidance may disrupt rhythmicity and contribute to increased gait asymmetry. These conclusions suggest that while RAC can enhance gait symmetry in certain conditions, their efficacy and potential disturbances vary across different walking contexts and neurological statuses.

## Data Availability

The original contributions presented in the study are included in the article/Supplementary material, further inquiries can be directed to the corresponding author.
