# Supplementary material for: Rhythmic auditory cues improve gait asymmetry during unobstructed walking in people with Parkinson’s disease but have no effect on obstacle avoidance - AsymmGait-Parkinson study
Source: Front Aging Neurosci. 2025 Feb 27;17:1455432. doi: 10.3389/fnagi.2025.1455432 (PMC11905297; doi:10.3389/fnagi.2025.1455432)
Supplement: Supplementary file 2 [file Data_Sheet_2.pdf]

This readme file was generated on [2024-03-16] by [JONATAS A CURSIOL]

GENERAL INFORMATION

Title of Dataset: Raw-data_Auditory-cue_Walking

Author/Principal Investigator Information

Name: Jônatas Augusto Cursiol

ORCID: 0000-0002-3250-5025

Institution: São Paulo State University (Unesp), Department of Physical Education, Human Movement Research Laboratory (MOVI-LAB), Bauru, Brazil

Address: Av. Eng. Luiz Edmundo C. Coube 14-01 - Núcleo Habitacional Presidente Geisel - Bauru - SP/SP - CEP 17033-360.

Email: jonatas.cursiol@unesp.br

Author/Associate or Co-investigator Information

Name: Fabio Augusto Barbieri

ORCID: 0000-0002-3678-8456

Institution: São Paulo State University (Unesp), Department of Physical Education, Human Movement Research Laboratory (MOVI-LAB), Bauru, Brazil

Address: Av. Eng. Luiz Edmundo C. Coube 14-01 - Núcleo Habitacional Presidente Geisel - Bauru - SP/SP - CEP 17033-360.

Email: fabio.barbieri@unesp.br

Information about funding sources that supported the collection of the data: Grant #2012/24040-9, São Paulo Research Foundation (FAPESP).

SHARING/ACCESS INFORMATION

Licenses/restrictions placed on the data: Not applicable.

Links to publications that cite or use the data: Not applicable.

Links to other publicly accessible locations of the data: Not applicable.

Links/relationships to ancillary data sets: Not applicable.

Was data derived from another source? Not applicable.

DATA & FILE OVERVIEW

File List: For this DATASET, there is only one associated file, in csv. format, called "Raw-data_Auditory-cue_Walking". It contains information on kinematic gait parameters raw data obtained during unobstructed and obstacle avoidance walking with auditory cues of people with Parkinson’s disease and neurologically healthy individuals.

Are there multiple versions of the dataset? No.

METHODOLOGICAL INFORMATION

Purpose of the test campaign

The aim of the study was to investigate the influence of auditory cues on gait asymmetry during unobstructed and obstacle avoidance walking in people with Parkinson's disease.

Protocol

The data collection protocol, which was performed in a single session, included an interview to collect clinical data, medication and disease diagnosis, as well as gait assessments. Five trials in each auditory cue condition were performed in unobstructed walking and 10 trials in each auditory cue condition - five for each limb crossing the obstacle - in obstacle avoidance during walking. The acquisition of kinematic gait parameters was accomplished by GAITRite® (CIR System, Clifton, NJ, USA) using a sample rate of 200 samples/s. For unobstructed gait, the steps (one for each side) in the middle of the pathway were analyzed.

For obstacle avoidance, the step during obstacle avoidance was analyzed. The step length, duration, width, and speed, limb swing duration (percentage of step duration), and double support duration (percentage of step duration) were calculated for each step. To investigate the gait asymmetry, we considered the steps with the most and least affected limb (right or left according to UPDRS items) for Parkinson disease participants as well as preferred and non-preferred limb (right or left according to footedness test) for the control group in each gait type.

DATA-SPECIFIC INFORMATION FOR: Raw-data_Auditory-cue_Walking

Number of variables: Step, from gait analyses: six (length, duration, width, velocity, limb swing duration and double support duration). For each of these six variables, information regarding the preferred and non-preferred limb was extracted in the conditions with an unobstructed path and path with an obstacle, as well as in the conditions with and without an auditory cue.

Number of cases/rows: 48 (eight columns for each variable, one of each associated with the information extracted from the preferred or least affected limb unobstructed, preferred or least affected limb with obstacle, non-preferred or most affected limb unobstructed, non-preferred or most affected limb with obstacle, preferred or least affected limb unobstructed and with auditory cue, preferred or least affected limb with obstacle and with auditory cue, non-preferred or most affected limb unobstructed and with auditory cue and non-preferred or most affected limb with obstacle and with auditory cue).

Variable List:

Column A - Group : the first 13 rows for data referring to participants in the control group (CG) and the final 13 rows for participants with Parkinson's disease (PD)

Column B - SL_pre_U : step length of the preferred or least affected limb unobstructed in centimeters

Column C - ST_pre_U : step duration of the preferred or least affected limb unobstructed in seconds

Column D - SW_pre_U : step width of the preferred or least affected limb unobstructed in centimeters

Column E - SV_pre_U : step velocity of the preferred or least affected limb unobstructed in centimeters per second

Column F - SW%_pre_U : percentage of limb swing duration of the preferred or least affected limb unobstructed in percentage of step duration

Column G - DS%_pre_U : percentage of double support duration of the preferred or

least affected limb unobstructed in percentage of step duration

Column H - SL_npre_U : step length of the non-preferred or most affected limb unobstructed in centimeters

Column I - ST_npre_U : step duration of the non-preferred or most affected limb unobstructed in seconds

Column J - SW_npre_U : step width of the non-preferred or most affected limb unobstructed in centimeters

Column K - SV_npre_U : step velocity of the non-preferred or most affected limb unobstructed in centimeters per second

Column L - SW%_npre_U : percentage of limb swing duration of the non-preferred or most affected limb unobstructed in percentage of step duration

Column M - DS%_npre_U : percentage of double support duration of the non-preferred or most affected limb unobstructed in percentage of step duration

Column N - SL_pre_O : step length of the preferred or least affected limb with obstacle in centimeters

Column O - ST_pre_O : step duration of the preferred or least affected limb with obstacle in seconds

Column P - SW_pre_O : step width of the preferred or least affected limb with obstacle in centimeters

Column Q - SV_pre_O : step velocity of the preferred or least affected limb with obstacle in centimeters per second

Column R - SW%_pre_O : percentage of limb swing duration of the preferred or least affected limb with obstacle in percentage of step duration

Column S - DS%_pre_O : percentage of double support duration of the preferred or least affected limb with obstacle in percentage of step duration

Column T - SL_npre_O : step length of the non-preferred or most affected limb with obstacle in centimeters

Column U - ST_npre_O : step duration of the non-preferred or most affected limb with obstacle in seconds

Column V - SW_npre_O : step width of the non-preferred or most affected limb with obstacle in centimeters

Column W - SV_npre_O : step velocity of the non-preferred or most affected limb with obstacle in centimeters per second

Column X - SW%_npre_O : percentage of limb swing duration of the non-preferred or most affected limb with obstacle in percentage of step duration

Column Y - DS%_npre_O : percentage of double support duration of the

non-preferred or most affected limb with obstacle in percentage of step duration

Column Z - SL_pre_ac_U : step length of the preferred or least affected limb unobstructed and with auditory cue in centimeters

Column AA - ST_pre_ac_U : step duration of the preferred or least affected limb unobstructed and with auditory cue in seconds

Column AB - SW_pre_ac_U : step width of the preferred or least affected limb unobstructed and with auditory cue in centimeters

Column AC - SV_pre_ac_U : step velocity of the preferred or least affected limb unobstructed and with auditory cue in centimeters per second

Column AD - SW%_pre_ac_U : percentage of limb swing duration of the preferred or least affected limb unobstructed and with auditory cue in percentage of step duration

Column AE - DS%_pre_ac_U : percentage of double support duration of the preferred or least affected limb unobstructed and with auditory cue in percentage of step duration

Column AF - SL_npre_ac_U : step length of the non-preferred or most affected limb unobstructed and with auditory cue in centimeters

Column AG - ST_npre_ac_U : step duration of the non-preferred or most affected limb unobstructed and with auditory cue in seconds

Column AH - SW_npre_ac_U : step width of the non-preferred or most affected limb unobstructed and with auditory cue in centimeters

Column AI - SV_npre_ac_U : step velocity of the non-preferred or most affected limb unobstructed and with auditory cue in centimeters per second

Column AJ - SW%_npre_ac_U : percentage of limb swing duration of the non-preferred or most affected limb unobstructed and with auditory cue in percentage of step duration

Column AK - DS%_npre_ac_U : percentage of double support duration of the non-preferred or most affected limb unobstructed and with auditory cue in percentage of step duration

Column AL - SL_pre_ac_O : step length of the preferred or least affected limb with obstacle and with auditory cue in centimeters

Column AM - ST_pre_ac_O : step duration of the preferred or least affected limb with obstacle and with auditory cue in seconds

Column AN - SW_pre_ac_O : step width of the preferred or least affected limb with obstacle and with auditory cue in centimeters

Column AO - SV_pre_ac_O : step velocity of the preferred or least affected limb with obstacle and with auditory cue in centimeters per second

Column AP - SW%_pre_ac_O : percentage of limb swing duration of the preferred or least affected limb with obstacle and with auditory cue in percentage of step duration

Column AQ - DS%_pre_ac_O : percentage of double support duration of the preferred or least affected limb with obstacle and with auditory cue in percentage of step duration

Column AR - SL_npre_ac_O : step length of the non-preferred or most affected limb with obstacle and with auditory cue in centimeters

Column AS - ST_npre_ac_O : step duration of the non-preferred or most affected limb with obstacle and with auditory cue in seconds

Column AT - SW_npre_ac_O : step width of the non-preferred or most affected limb with obstacle and with auditory cue in centimeters

Column AU - SV_npre_ac_O : step velocity of the non-preferred or most affected limb with obstacle and with auditory cue in centimeters per second

Column AV - SW%_npre_ac_O : percentage of limb swing duration of the non-preferred or most affected limb with obstacle and with auditory cue in percentage of step duration

Column AW - DS%_npre_ac_O : percentage of double support duration of the non-preferred or most affected limb with obstacle and with auditory cue in percentage of step duration
